# Physiological Dynamics in Demyelinating Diseases: Unraveling Complex Relationships through Computer Modeling

**DOI:** 10.3390/ijms160921215

**Published:** 2015-09-07

**Authors:** Jay S. Coggan, Stefan Bittner, Klaus M. Stiefel, Sven G. Meuth, Steven A. Prescott

**Affiliations:** 1 NeuroLinx Research Institute, La Jolla, CA 92039, USA; E-Mail: klaus@neurolinx.org; 2 Department of Neurology, Institute of Physiology, Universitätsklinikum Münster, 48149 Münster, Germany; E-Mails: stefan-bittner@outlook.com (S.B.); sven.meuth@ukmuenster.de (S.G.M.); 3 Neurosciences and Mental Health, the Hospital for Sick Children, Toronto, ON M5G 1X8, Canada; E-Mail: steve.prescott@sickkids.ca; 4 Department of Physiology and the Institute of Biomaterials and Biomedical Engineering, University of Toronto, Toronto, ON M5G 1X8, Canada

**Keywords:** myelin, demyelination, multiple sclerosis, neurodegenerative disease, computational model, drug discovery

## Abstract

Despite intense research, few treatments are available for most neurological disorders. Demyelinating diseases are no exception. This is perhaps not surprising considering the multifactorial nature of these diseases, which involve complex interactions between immune system cells, glia and neurons. In the case of multiple sclerosis, for example, there is no unanimity among researchers about the cause or even which system or cell type could be ground zero. This situation precludes the development and strategic application of mechanism-based therapies. We will discuss how computational modeling applied to questions at different biological levels can help link together disparate observations and decipher complex mechanisms whose solutions are not amenable to simple reductionism. By making testable predictions and revealing critical gaps in existing knowledge, such models can help direct research and will provide a rigorous framework in which to integrate new data as they are collected. Nowadays, there is no shortage of data; the challenge is to make sense of it all. In that respect, computational modeling is an invaluable tool that could, ultimately, transform how we understand, diagnose, and treat demyelinating diseases.

## 1. Introduction

The nervous systems of vertebrates are often divided into grey and white matter based on their appearance and corresponding functional roles. While the grey matter consists largely of cell bodies and dendrites, white matter contains mostly axons and gets its name from the lipid membrane sheets called myelin that are wound tightly around those axons [1]. Myelin originates from different classes of glial cells referred to as oligodendrocytes in the central nervous system (CNS) and Schwann cells in the peripheral nervous system (PNS).

The electrical insulation provided by the myelin sheets improves axonal function by increasing both the energy efficiency and conduction velocity of action potentials (APs). These two functions may have switched their relative importance during evolution [2]. Myelin first appeared in the Ordovician (485 to 443 ma, or million years before the present) after the split of lamprey and hagfish ancestors from the remainder of the vertebrate lineages [3]. With some interesting exceptions [4,5], myelin or analogous structures are found in all vertebrates and is critical for the proper functioning of their nervous systems. The approximate time of the evolution of myelin can be deduced from the known time of divergence between chordates without (agnatha) and with (all other vertebrates) myelin.

The myelin wrapping is interrupted by regularly spaced, un-myelinated stretches known as the nodes of Ranvier. Myelin speeds up conduction by restricting transmembrane charge flow through ion channels located within the nodes. Within the so-called internodes, current flows down the axon with little of it passing across the insulated cell membrane. The AP is regenerated at each node where the density of voltage-gated sodium and potassium channels is very high. This process is called “saltatory conduction” since the AP seems to jump from node to node. Disruptions in this rapid-fire communications system can be associated with an array of nervous systems dysfunctions [6].

In several regards, axons appear to operate at physical limits. One interesting example is that the size of axons seems to be constrained by the thermal noise intrinsic to ion channel proteins; any axon thinner than 0.1 μm would be useless for information transfer due to its high noise levels [7]. Intriguingly, 0.1 μm is also roughly the smallest axon diameter observed in nervous systems [7]. This and similar findings suggest that axons and their substructures are finely tuned biological devices, but that tuning can evidently be disrupted under pathological conditions [8].

Demyelination sets in motion functional changes that are important for clinical features but which are not readily explained by immunological or radiological changes. Location of a plaque predicts what system will be affected (motor *vs*. sensory, visual *vs.* tactile) but not how it will be affected. This highlights the importance of assessing function (in addition to structure) and how it changes following demyelination. After introducing demyelinating diseases, we will discuss how the clinical manifestations of those diseases reflect diverse pathological changes in axon function. We will argue that understanding those changes and fully capitalizing on that understanding for diagnostic and therapeutic purposes can benefit enormously from computational modeling.

## 2. Demyelinating Diseases

**Figure 1:**
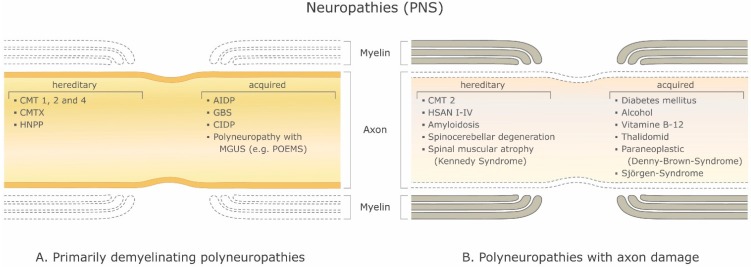
Demyelinating disorders of the peripheral nervous system (PNS). (**A**) Primary demyelinating polyneuropathies and (**B**) Polyneuropathies with axon damage. Abbreviations: CMT 1, 2 and 4: Charcot-Marie-Tooth disease; CMTX: X-linked Charcot-Marie-Tooth disease; HNPP: hereditary neuropathy with liability to pressure palsies; AIDP: acute inflammatory demyelinating polyneuropathy; GBS: Guillain-Barré syndrome. CIDP: chronic inflammatory demyelinating polyneuropathy; MGUS: monoclonal gammopathy of undetermined significance; POEMS: polyneuropathy, organomegaly, endocrinopathy or edema M-protein and skin abnormalities; HSAN I–IV: hereditary sensory and autonomic neuropathy.

**Figure 2:**
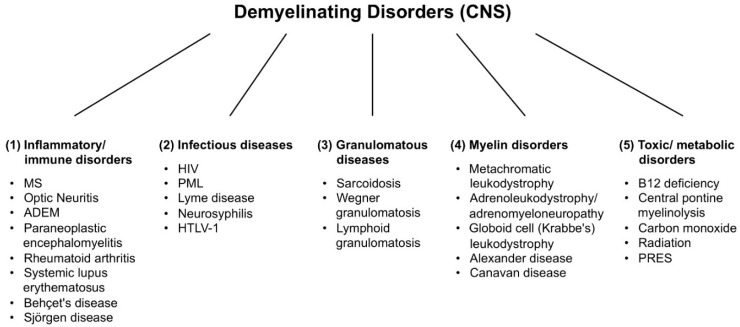
Demyelinating disorders of the central nervous system (CNS). Abbreviations: MS: multiple sclerosis; ADEM: acute disseminated encephalomyelitis; HIV: human immunodeficiency virus; PML: progressive multifocal leukoencephalopathy; HTLV-1: human T-lymphotropic virus 1; PRES: posterior reversible encephalopathy syndrome.

There are a large number of demyelinating diseases affecting both the PNS (Figure 1) and CNS (Figure 2). The etiologies are heterogeneous, ranging from genetic disorders to metabolic, infectious or autoimmune mechanisms. Multiple sclerosis (MS) is the most prevalent of these disorders, with an estimated 3 million patients worldwide. Its underlying cause is uncertain but is thought to involve genetic predisposition to environmental agents [9,10] and can involve immunological, responsiveness to trauma, biophysical, genetic and/or metabolic components [10]. The symptoms and lesions must be multiple in both time and space. That is, there must be multiple episodes in time, involving disconnected parts of the central nervous system. It is not clear whether inflammatory demyelination is a primary or secondary event within the disease process [9,11,12]. Most treatments target the immune system or the blood-brain barrier, but managing neurological symptoms through modulation of axonal excitability also plays an important role (see below).

### 2.1. Clinical Assessment of Multiple Sclerosis

Symptoms are diverse and can occur in all combinations within an individual patient. Diagnosis requires that there must be multiple lesions and symptomatic episodes over time, involving disconnected parts of the CNS. Furthermore, symptoms tend to be poorly correlated with radiological measures. In the great majority of cases, individual clinical characteristics do not correlate well with MRI findings, especially for cerebral lesions [13,14,15]. This clinico-radiologic dissociation begs for better theoretical understanding of demyelination symptoms and the underlying biophysical changes that accompany them, which of course raises the question of what exactly happens to the affected axons.

Symptoms are often intermittent and can include both loss-of-function (negative symptoms such as numbness, muscle weakness, tingling, blindness, incontinence, loss of sexual function, loss of balance, slurred speech, constipation, disabling fatigue, depression, cognitive dysfunction, inability to swallow, gait disruption and loss of breathing control) and gain-of-function (positive symptoms such as spasms, spasticity, cramps, pain, blurred or double vision, urinary urgency or hesitancy, nausea, among others) [16]. Early differential diagnostic criteria include Lhermitte’s sign (neck flexion-related sensations) and Uhthoff’s phenomenon (temperature-dependent worsening of symptoms). The differential diagnosis of MS closely follows the McDonald criteria [17].

In human diagnostic studies of visual, sensory or motor evoked potentials (VEP, SEP, MEP), only latency or conduction velocity can be measured accurately (with approximately 30%–40% variations between different laboratories). But these measures give little clue about underlying mechanisms that involve conduction slowing or block, or morphological or functional factors such as branching, demyelination, remyelination, axonal tapering (decrease in cross-sectional area), attenuation or regrowth, temperature-related changes in conduction, or malpolarization (hyper or hypo). Nonetheless, the type of demyelinating lesion can yield clues regarding etiology and therefore guide treatment; for example, genetic factors seem to more strongly correlate to internodal disease processes and immunological dysfunctions cause paranodal abnormalities [18].

A number of tests are routinely used to assess the neural function. In electroneurography, a brief electrical stimulus is applied to a peripheral nerve at an anatomically predefined position in order to measure the latency and amplitude of the compound action potential at another location along the nerve. Results need to be interpreted in combination with clinical findings and tests (e.g., electromyography) but, importantly, different diseases exhibit different patterns of electroneurographic changes. This is important not only for diagnostic purposes but can also point to specific pathological changes in axon function which could, in turn, help guide the choice of therapy (if the axon pathobiology were understood; see below). Using threshold tracking, excitability has been measured in humans for the several peripheral demyelinating diseases including Charcot-Marie-Tooth Disease Type 1A (CMT1A), chronic inflammatory demyelinating polyneuropathy (CIDP), Guillain-Barré syndrome (GBS) and multifocal motor neuropathy (MMN) [19,20,21,22,23,24,25]. The challenge lies in interpreting those observations. To this end, the group of Stephanova has simulated progressively greater degrees of systematic and focal demyelination of motor fibers to try to explain the observed physiological changes [26,27,28,29,30,31] (see Modeling section below).

### 2.2. Involvement of Cell Bodies

Progression from relapsing-remitting MS (RRMS) to the secondary progressive MS (SPMS) is associated with greater involvement of grey matter pathology, although axonal/grey matter involvement can be observed already in early disease stages [32,33,34,35]. Damage to the grey matter is regarded as the underlying mechanism of disease progression and permanent disability in MS patients, and is measured by loss of brain parenchymal fraction or brain volume by MRI or clinically by progression on the expanded disability status scale (EDSS) [36]. Transition from RRMS to SPMS is foreboding for the lack of therapeutics to combat the exacerbated physical and cognitive deterioration that most SPMS patients face [9,37].

### 2.3. Treatment

The main interventions for MS involve modulation of the immune response with, for example, methly-prednisolone, interferon betas, glatiramer acetate or fingolimod, or by preventing inflammatory cells from crossing the BBB (monoclonal antibodies e.g., Tysabri (anti α4-integrin, Natalizumab)). Very recently the first two oral agents (fumarate and teriflunomide) as well as the anti-CD52 directed antibody Natalizumab where approved for treatment of RRMS, which can be successfully treated by first-line therapies like interferons, glatiramer acetate, or fingolimod, or by second-line therapies, but progressive forms (PPMS, SPMS) still represent an unmet biomedical need [38]. Anti-neoplastics are used in extremely advanced or difficult cases [39].

**Figure 3:**
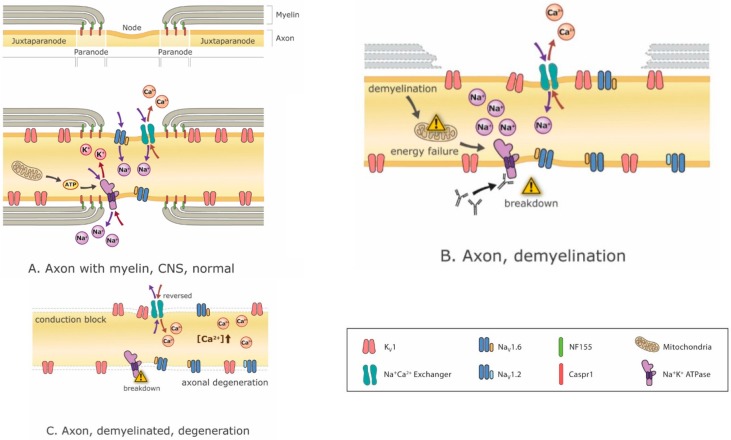
Mechanisms of demyelination-related Neurodegeneration. Demyelination can result prorgressively in ionic disequilibria, energy crisis, conduction block and eventually neurodegeneration. (**A**) a normal node of Ranvier with juxtaparanodal, paranodal and nodal regions intact, depicting Na^+^, K^+^ and Ca^2+^ ions flowing through their respective channels with mitochondria supplying the ATP for energy-dependent Na^+^K^+^ ATPases that re-establish the ion gradients depleted by ion flux through channels. Numerous different ion channels are present in the axon but only a small subset is depicted here; (**B**) partial demyelination results in dispersal of nodal ion channels, energy insufficiency and disequilibria of ion gradients; (**C**) complete demyelination can result in conduction block and axonal degeneration due to the accumulation of intracelluar Ca^2+^ that results from energy crisis and disruption of ionic balances. Abbreviations: K_v_1: potassium channel type 1; Na_v_1.6 and Na_v_1.2: sodium channel types 1.6 and 1.2; Na+ Ca+ Exchanger: Na-Ca exchange pump; Na^+^K^+^ ATPase: ATP (energy)-dependent Na-K exchange pump; CASPR1: contactin-associated protein 1 (interaction molecule between myelinating cell with axon); NF155: neurofascin 155 (predominant interaction molecule between myelin and axon at paranodal axo-glial junction).

Disease-modifying drugs are critical for stopping or at least attenuating the demyelination process, but so too is it critical to manage the symptoms arising from whatever demyelination has already occurred. Ion channel modulation is increasingly promising with the advent of new ion channel blockers such as Ampyra (K-channel blockade) [40,41]. Potassium channel blockade is intended to enhance axon excitability. The problem is that such interventions, while effective in treating negative symptoms and restoring function, tend to exacerbate positive symptoms [42]. Conversely, treating positive symptoms such as spasms with anti-epileptics like carbamazepine, for example, can exacerbate negative symptoms [43]. In fact, blocking Na^+^ channels not only reduces positive symptoms, it may also be neuroprotective (because Na^+^ accumulation causes Na^+^/Ca^2+^ exchange mechanisms to load neurons with Ca^2+^, which is excitotoxic) [44] (Figure 3) but these benefits come at the expense of negative symptoms. Therefore, and especially in a patient exhibiting a mixture of positive and negative symptoms, treatment options are restricted.

The above discussion raises the important point that although much ado has been made about immune mechanisms, their connection with clinical changes is largely correlational. One must consider the intermediary effects on axonal function, namely the primary and secondary (compensatory) changes in axon excitability, in order to appreciate how neurological function is altered. Those changes are not simple and direct consequences of demyelination but, instead, suggest that axonal physiology itself changes in response to demyelination. Some of those changes are adaptive whereas others are maladaptive, or perhaps adaptive changes can become maladaptive as the situation (myelination status) evolves. If changes in axonal physiology dictate the manifestation of various symptoms, then symptom management will largely fall on treatments that aim to manipulate axon physiology. Strategically developing such treatments require a deep, mechanistic understanding of axonal excitability and its regulation.

## 3. Axon Pathobiology

### 3.1. Structural and Molecular Changes

Axons are profoundly affected by demyelination. Axon morphology becomes irregular or swollen, often with a beaded appearance. Focal accumulation of proteins (by fast axonal transport) is also observed. In chronic active plaques, axonal loss of 20%–80% is apparent within peri-plaque white matter and normal distant white matter [45]. In early active and chronic active plaques, damage is thought to be caused by inflammatory and immune factors released during acute inflammatory demyelination. Proposed mediators include proteases, cytokines, excitotoxins and free radicals. Neuronal antigens are targets of immune reaction leading to CNS inflammation. Other factors causing axonal dysfunction or death include a lack of trophic support from myelin and oligodendrocytes, damage from soluble or cellular immune factors still present in the inactive plaque, and chronic mitochondrial failure in the setting of increased energy demands [46]. A critical role for oligodendrocytes and Schwann cells in axon survival has also been attributed to peroxisomes, lipid metabolism and reactive oxygen species (ROS) detoxification [47].

Remyelination is often observed as shadow plaques formed by the recruitment of undifferentiated oligodendrocyte precursors that migrate to and surround the lesions enabling thin layers of remyelination [48]. This process occurs mostly in acute active plaques, but also in chronic phases. This observation triggered the development of a new monoclonal anitbody directed against LINGO-1 (Anti-LINGO-1). Binding of LINGO-1 to Nogo receptors prevents remyelinating processes in the CNS; inhibition of this interaction thus enables significant remyelination in animals with experimental autoimmune encephalomyelitis [49].

During the disease process, autoreactive lymphocytes and macrophages can cross the blood brain barrier and accumulate in the brain and spinal cord [50]. Regulatory lymphocytes (Tregs) fail to suppress effector cells-mostly cytotoxic CD8^+^ cells [51]. Release of pro-inflammatory cytokines recruits naive microglia, which make contact with an oligodendrocyte-myelin unit by interactions with Fc and complement receptors. A cytotoxic death-triggering signal is then transmitted through surface bound tumor necrosis factor α (TNFα) [52]. This occurs in concert with extensive axonal damage [10].

Lucchinetti *el al*. [46] proposed four distinct immunopatterns of plaque formation found in patients at different stages of the disease. Type I and II plaques are dominated by T-lymphocyte and macrophage inflammation and are thought to mimic T-cell or T-cell plus antibody autoimmune encephalomyelitis models, respectively. Myelin loss in type I plaques may be caused by toxic factors released by activated macrophages, whereas IgG and complement deposition suggest a role of antibodies in type II plaques. In contrast, patterns III and IV show large oligodendrocyte dystrophy. Pattern III is thought to be related to hypoxia-induced lesions which are driven by defects in mitochondrial function [53], whereas pattern IV lesions are associated with profound non-apoptotic death of oligodendrocytes in periplaque white matter.

Barnett and Prineas [54] analyzed lesions from patients directly after the onset of a relapse, during which active plaque formation was ongoing. Their results suggest that oligodendrocyte apoptosis and glial activation occur during early active plaque formation in the absence of inflammatory lymphocytes or myelin phagocytes. They proposed that the vulnerability of oligodendrocytes, described in Lucchinetti’s type III pattern, is present in the early stages of all plaque formation and is the trigger for subsequent post apoptotic necrosis which initiates the phagocytosis of myelin by macrophages at later stages. In vitro analyses of this process have implicated complement cascades, tumor necrosis factors or gaseous second messengers [55]. Although identification of plaques and monitoring of their progress has important clinical value, there is only a modest correlation between the demyelinating lesion load as determined by conventional MRI and the clinical disability of patients with MS (see above).

### 3.2. Functional Changes

The mechanisms of functional impairment during demyelination often include the disruption of transmembrane Na^+^, K^+^ and Ca^2+^ ions, the dispersal of their corresponding ion channels, a decrease in the efficiency of AP conduction and a resulting metabolic crisis (Figure 3). Demyelination can readily explain conduction failure within the affected axon. If conduction does not completely fail, conduction velocity can nonetheless be slowed and differential slowing across different axons can cause variable conduction delays that result in desynchronized spiking.

Demyelination also allows denuded axons to become closely apposed, thus setting the stage for ephaptic interactions and crosstalk [10]. Reflection can also occur because of impedance mismatch between myelinated and unmyelinated lengths of axon. On the other hand, hyperexcitability cannot be directly ascribed to demyelination; instead, secondary changes in intrinsic excitability need to be invoked to explain phenomena like ectopic spike generation and afterdischarge (AD). Alterations in excitability likely represent compensatory changes aimed at restoring function following the disruption caused directly by demyelination, consistent with a process referred to as homeostatic plasticity [56], but that compensation can evidently be maladaptive. Each of the aforementioned outcomes, which are not mutually exclusive, contribute to producing different symptoms observed in demyelinating diseases.

Paroxysmal symptoms characterized by the sudden onset or intensification of symptoms such as spasm or shooting pain likely arise from AD or otherwise inappropriate burst-type spiking. Such spiking patterns suggest highly nonlinear interactions among the contributing ion currents [57,58] and could, in theory at least, involve interactions between different regions of the neuron [59]. As opposed to more generic forms of hyperexcitability (e.g., increased firing rate or reduced threshold), these specific patterns are limited in terms of the precise mechanisms through which they might arise. Therefore, identifying the ion channel changes underlying those specific forms of hyperexcitability can help constrain the search for ion channel changes responsible for associated, yet less distinctive, forms of hyperexcitability.

The disruption of energy balance in a neuron could also profoundly impact neuron well-being (Figure 3). Indeed, compensatory changes may manage to restore certain functions but, without reversing the primary problem, other problems may arise. For example, even if conduction block is prevented by an appropriate compensatory change in excitability (*i.e*., one that does not result in hyperexcitability), the system may be less energy efficient. Losing the energy savings afforded by saltatory conduction induces compensatory mitochondrial energy production that can result in oxidative damage and neurodegeneration [53,60,61].

Keeping track of this long list of neurobiological changes, understanding the inter-relationships between those changes, and ultimately linking those changes with clinical manifestations and applying effective treatment is no easy task. To this end, computational modeling is an invaluable tool. Simulations not only serve to organize what information is already known, they also identify crucial gaps in knowledge. The judicious use of computational modeling can therefore enable more comprehensive understanding and facilitate the more effective application of that understanding, as discussed below.

## 4. Computational Modeling

Especially when paired with traditional experiments, computational modeling is indispensable for making sense of inconsistent data and complex mechanisms. These benefits are exemplified by the application of simulations in other fields, such as epilepsy [62]. Here we survey some of the history of computational modeling of axons, ion conductances, the physiology of myelin and demyelination, the immune system, mitochondria and other biological factors that are critical for understanding demyelinating diseases. Our review is not exhaustive but will provide a broad introduction to past, present, and future efforts in this area.

### 4.1. Modeling Axons

The computational modeling of axons has evolved taxonomically, from squid to mammalian tissues with a corresponding increase in sophistication. The Hodgkin and Huxley (HH) model, which provided the first thorough explanation of AP generation, was derived from experiments in unmyelinated giant axons of squid [63,64], but this early model has proven to be an invaluable tool from which later, more sophisticated models of myelinated axons have evolved.

The spatial and biophysical heterogeneity conferred by the addition of myelin, and the consequent formation of nodes and internodal regions, represents a significant increase in axon complexity. The first computational model of a myelinated axon was a one-dimensional model that collapsed the myelin sheath into the underlying passive axolemma, used a uniform spatial step size to form the discrete approximation used in the numerical solution and employed a HH characterization of the nodal membrane [65]. Goldman & Albus [66] modified this model to include a description of the nodal membrane derived from experimental data on Xenopus laevis myelinated nerve fibers as determined by Frankenhaeuser & Huxley [67]. Subsequent studies have used the same basic form for the model with some variations for the representation of the axolemma [15,68,69,70,71,72,73,74,75,76]. The single cable model, describing the axon and all of its conductance and capacitance properties in one cable equation, has dominated the field until the present day despite the introduction of double cable models by Blight [77]. In double cable models, the internodal axolemma and the myelin sheath are independently represented. The double cable model has been expanded by Halter and Clark [78] to explore effects of the complex geometry of CNS oligodendrocytes (or Schwann cells in the case of the PNS).

Newer models have also improved upon previous simplifications including the anatomical complexity of the node of Ranvier, the distribution of ionic channels in the axon beneath the myelin sheath, the different electrical properties of the myelin sheath and the axolemma, and accommodation of possible current flow within the periaxonal space [78,79,80,81,82]. Anatomical representations of the paranodal area have allowed more detailed assessment of the effects of traumatic brain injury (TBI) on myelinated axons [83]. One of the most anatomically sophisticated models includes representation of the complex aqueous sheath structure of myelin lamellae as a series of interconnecting parallel lamellae in a model of motor nerves [30,80].

Newer models have also considered the non-uniform distribution of ion channels throughout the axon [19,84,85,86,87,88,89,90]. Beyond ion channels, energy-dependent pumps and other ion-transport mechanisms provide important therapeutic targets for a number of neurological disorders [91,92,93]. In that respect, regulating transmembrane ion gradients costs significant energy and itself becomes an important consideration (see below) [94]. This is especially true since the small volume of axons renders them prone to ion concentration changes that can dramatically impact driving forces, and can become problematic in models that assume constant intracellular and extracellular concentrations. But recent models have also dealt with such issues (see below).

All of the aforementioned models focus on simulating the change in axon membrane potential but one does not necessarily have experimental access to that variable, which of course complicates efforts to compare simulation and experimental data. Indeed, since extracellular recordings are the primary source of electrophysiological data from human subjects, the mathematical description of the extracellular field potential is of great interest clinically. Mathematical evaluations based on Laplace equations and Fourier transforms are used for calculating these potentials (sometimes referred to as line-source modeling, e.g., [82,95]).

### 4.2. Modeling Specific Mechanisms

Beyond modeling normal axonal function, models can be used to explore particular mechanisms of axonal dysfunction especially when combined with experimental results that might better pinpoint mechanisms [96]. For example, Barrett and Barrett [97] showed that the depolarizing afterpotential (DAP) is sensitive to changes in conductance densities and capacitative changes that might occur during demyelination. A model by Blight was designed for simulation of his experimental recording conditions [77,98] and represents a single internode with multiple discrete segments and adjacent nodes and internodes in single lumped-parameter segments. This model included K^+^ channels in the axolemma of the single multi-segmented internode and treats the remainder as purely passive.

Building on this work, with careful attention to anatomical and electrophysiological details, McIntyre *et al*. [81] addressed the role of the DAP and afterhyperpolarization (AHP) in the recovery cycle—the distinct pattern of threshold fluctuation following a single action potential exhibited by human nerves. The simulations suggested distinct roles for active and passive Na^+^ and K^+^ channels in both afterpotentials and proposed that differences in the AP shape, strength-duration relationship, and the recovery cycle of motor and sensory nerve fibers can be attributed to kinetic differences in nodal Na^+^ conductances. Richardson *et al.* [99] also found that alteration to the standard “perfect insulator” model is necessary to reproduce DAPs during high-frequency stimulation.

The temperature sensitivity of demyelination effects has also been investigated computationally. Zlochiver [100] modeled persistent resonant reflection across a single focal demyelination plaque and found that this effect was sensitive to temperature and axon diameter. All of these examples demonstrated the power of simulations to examine specific mechanisms to explain observed phenomena from the clinic and offer guidance for future research.

As mentioned above, distinct changes in axon function are likely to manifest certain gain- or loss-of-function symptoms. If one could reproduce those changes in a computational model, the necessary parameter changes needed to convert the model between normal and abnormal operation could be used to predict the underlying pathology. Ideally this can lead to specific experiments in which the suspect ion channel, for example, is directly manipulated to see if its acute alteration is sufficient to reproduce or reverse certain pathological changes. Recent studies from the Prescott lab illustrate this process [101,102]. This success of these studies depended on advanced techniques including the dynamic clamp technique, used to switch between normal and abnormal spiking patterns and optogenetic tools. The next step is to link changes in axon function with disease symptoms (or their behavioural correlates in animal models).

In auditory nerve experiments, Tagoe and colleagues [103] demonstrated that hearing loss related to morphological changes at paranodes and juxtaparanodes, including the elongation of the auditory nerve around nodes of Ranvier, can result from exposure to lound noise, Extending this work, Hamann and collegues built a computational model to examine possible mechanisms. Their model suggested that it is more likely that a decrease in the density of Na-channels, rather than a redistribution of Na or K channels in general, is responsible for the conduction inhibition associated with acoustic over-exposure [104]. This experiment-model tandem demonstrates the revelatory potential of pairing computational models with laboratory experiments.

With a myelinated axon multi-layered model Stephanova and colleagues have had on-going success identifying likely anatomical and physiological deficiencies underlying various symptoms and conditions related to demyelination by making comparisons to the threshold tracking measurements from patients including latencies, refractoriness (the increase in threshold current during the relative refractory period), refractory period, supernormality, and threshold electrotonus values including stimulus-response measures such as current-threshold relationships [21]. For example, they found that mild internodal systematic demyelination (ISD) is a specific indicator for CMT1A. Mild paranodal systematic demyelination (PSD) and paranodal systematic demyelination (PISD) are specific indicators for CIPD and its subtypes. Severe focal demyelinations, internodal and paranodal, paranodal-internodal (IFD and PFD, PIFD) are specific indicators for acquired demyelinating neuropathies such as GBS and MMN [18] (see Figure 1).

Mild systematic and severe focal demyelination correspond to hereditary (CMT1A) and acquired (CIDP, GBS and MMN) neuropathies (Table 1). It was also found that 70% systematic demyelination is insufficient to cause symptoms and 96% is required for conduction block at a single node [18]. Thus, there is a large safety factor for focal demyelination. With their temperature-dependent version of the model of the myelinated human motor nerve fiber, Stephanova and Daskalova [105] showed that the electrotonic potentials in patients with CIDP are in high risk for blocking during hypo- and even mild hyperthermia and suggest mechanisms involving increased magnitude of polarizing nodal and depolarizing internodal electrotonic potentials, inward rectifier K^+^ and leak K^+^ currents increase with temperature, and the accommodation to long-lasting hyperpolarization is greater than to depolarization.

**Table 1:** Correspondence between types of demyelination and diseases according to Stephanova and Dimitrov [18].

Type of Demyelination	Corresponding Disease (PNS)
Internodal systematic demyelination (ISD)	Charcot-Marie-Tooth Disease Type 1A (CMT1A)
Paranodal systematic demyelination (PSD)	Chronic inflammatory demyelinating polyneuropathy (CIDP)
Paranodal + internodal demyelination (PISD)	Chronic inflammatory demyelinating polyneuropathy (CIPD) subtypes
Internodal focal demyelination (IFD)	Guillain-Barré (GBS)
Paranodal focal demyelination (PFD)	Multifocal Motor Neuropathy (MMN)
Paranodal + focal demyelination (PIFD)	Multifocal Motor Neuropathy (MMN)

### 4.3. Simple Models and Nonlinear Dynamical Analysis

Given the temporal dissociation between the manifestation of symptoms and the rates of demyelination and remyelination, homeostatic processes undoubtedly occur within axons, which include the redistribution of ion channels in demyelinated plaques [106,107]. But given the diversity of ion channels expressed by different axons and only patchy knowledge of how expression levels change, building detailed models to investigate those homeostatic processes is problematic. Especially under those conditions, highly simplified models can help identify fundamental principles, as exemplified by joint use of modified HH and Morris-Lecar models [57,58]. The results of those studies suggested a simple explanation for the breadth of symptoms encountered during demyelination by revealing that the ratio of Na^+^ to leak K^+^ conductance, *g*(*Na*)/*g*(*L*), acted as a four-way switch controlling excitability patterns that included failure of AP propagation, normal AP propagation, AD, and spontaneous spiking.

Further studies with this model suggested the potential for competition or cooperation between different regions of the same neuron [59]. Cooperativity between remote sites of ectopic spiking allows AD to be initiated and maintained at different locations within a single axon, thus providing a compelling explanation for the temporal and spatial discontinuities of pain and other symptoms presented by MS patients. Remarkably, in a recent study of demyelinated axons in a cuprizone mouse model, experimental evidence was seen for a redistribution of ion channels from the node of Ranvier, enhanced ectopic excitability along with antidromically propagated APs from the demyelinated plaque, as well as a compensatory shift in the excitability of membranes proximal to the soma [108]. All of these observations concur or are consistent with the computational model predictions of Coggan and colleagues and imply the success of the computational approach to guiding laboratory studies.

Furthermore, these simplified models enabled application of mathematical tools to examine the nonlinear mechanisms by which AD is initiated and terminated [57,58,59]. Bifurcation analysis revealed the underlying bistability of axon excitability under pathological conditions, as well as the factors controlling the transition from one attractor state to another. AD, for example, requires a slow inward current that allows for two stable attractor states, one corresponding to quiescence and the other to repetitive spiking (a limit cycle). Termination of AD was explained by the attractor associated with repetitive spiking being destroyed. This occurred when ultra-slow negative feedback in the form of intracellular Na^+^ accumulation caused the destruction of the limit-cycle attractor state [58]. Other studies using bifurcation analysis suggest that ion concentration changes can introduce slow dynamics that may be important for understanding pathological outcomes [94,109].

### 4.4. Modeling at Small Scales

Studies mentioned above highlight the importance of ion concentration changes but each of them only considered those changes at a relatively course scale. By comparison, the study by Lorpreore *et al*. [110] tackled the daunting problem of modeling three-dimensional electro-diffusion of ion fluxes in micro and nano-domains surrounding ion channels at the node of Ranvier. In this unique model, the fluxes of ions are calculated by Poisson-Nernst-Planck equations with finite volume techniques. The fluxes and electric potentials were evaluated within voxels formed by a Delaunay-Voronoi mesh of the axon interior and exterior close to the membrane. Importantly, the algorithm was validated and results agreed with cable model predictions. Divergence from cable model predictions at smaller cluster sizes revealed the importance of each channel’s own electric field.

The above example highlights the point that models can simulate more than ion channels and membrane potential. Indeed, models can and must dig deeper into biophysical mechanisms like electro-diffusion and into signaling pathways that ultimately serve to regulate ion channel function and expression. A promising method called Biochemical Systems Theory (BST) may be useful in the future for pre-screening the effects of drugs at the systemic level. Broome and Coleman [111] demonstrated the power of this technique by modeling several biochemical pathways in neurons associated with cell death during MS including reactive oxygen and nitrogen species formation, Ca^2+^ dynamics, death complex formation, apoptotic factor release, and inflammatory responses together with three different states: normal, MS disease and treatment. At the atomic-level, a computational model of myelin basic protein (MBP) structure was carried-out because post-translational modifications of MBP may contribute to demyelination in MS [112]. It is important to understand its 3D structure to predict interaction sites with other molecules but a crystal structure for this protein might never be measured directly. This type of modeling may, therefore, represent an effective way to predict the structure by combining knowledge of amino acid sequence with information from similar proteins. The challenge for and the true power of modeling lies in connecting mechanisms that operate at vastly different scales, from molecular structure to the nervous system as a whole, and beyond, to address how the nervous system interacts with the immune system.

Models of Immune Factors. While there are numerous computational models of the immune system [113], those related to MS typically model genetic interaction networks, either represented as sets of ordinary differential equations (ODEs) or Boolean networks. One systems biology model of a possible cellular mechanism of RRMS found breakdown in homeostasis of effector (Teff) and regulatory T (Treg) cells [114,115]. By changing parameters in the Teff-Treg feedback loop, under continual stochastic external stimulus from antigens, the model reproduced spontaneous and apparently stochastic immune relapses. The irreversible damage from each episode accumulates over time. Novel predictions include the suggestion that the timing of Treg immunotherapy in the immune response cycle is critical in determining whether intervention is beneficial or deleterious.

Models of Mitochondrial Dysfunction. As mentioned above, myelin enables more energy efficient AP conduction along the axon. The increased energy demands placed on the demyelinated axon represents yet another challenge to the afflicted neuron. Beyond the loss of saltatory conduction, there is mounting evidence of a critical role for astrocytes and oligodendrocytes in supplying energy to neurons and this process has also been the subject of computational modeling [116].

There are many ways mitochondrial function can go awry and the compensatory pathways are equally complicated [53,60,61]. For example, mitochondrial dysfunction can be rooted in perturbed Ca^2+^ signaling within mitochondria, disrupted proton gradients or electron chain, reduction-oxidation imbalance as well as the consequences of reduced ATP availability, locally and globally. Multi-scale models of heart, for example, have been used to link altered mitochrondrial Ca^2+^ signaling to arrhythmia [60]. Using mitochondrial network modeling, this study demonstrated how even slightly too much reactive oxygen species can trigger a cell-wide collapse of mitochondrial membrane potential. This is an excellent example of how a computational model can link processes occurring at different levels, and it is precisely these linkages that must be established in the field of demyelination diseases.

## 5. Missing Links and the Need for Integration

Within the field of demyelinating diseases, modeling efforts have traditionally focused on axon models aimed at explaining various aspects of excitability. But as outlined above, those models have undergone tremendous evolution in complexity. In the process, models at different biological scales have begun to coalesce. For instance, models have now begun to address the regulation of ion concentrations and the consequences thereof for slow excitability changes, energy consumption, and toxicity. A computational approach will be necessary for integrating parallel and multifactorial etiologies associated with cognitive decline such as immune system signaling, energy metabolism, grey and white matter interactions, and genetic networks [117]. These continued efforts are starting to uncover the vast and interconnected feedback loops that operate across a broad range of spatial and temporal scales. That said, such efforts are still in their infancy and wide gaps remain in the modeling of demyelinating diseases. It is easier to describe what has been modeled than what has not. A truly integrated model involving multiple cell types that addresses all the hypothesized etiological factors remains unrealized. Among the unexplored or under-explored but potentially useful targets for modeling are grey matter pathology, myelin sheath aqueous layers, energy metabolism, and perhaps most importantly, multi-scale or integrated modeling. One should recognize that the necessary tools exist in other fields of study and can, therefore, be readily applied to the study of demyelination diseases.

## 6. Conclusions

The normal physiological function of the CNS or PNS relies on a highly regulated interplay of neurons, glia, vasculature and immune cells. This process encompasses and integrates numerous cellular and signaling components that produce a dynamical, computational whole. When any part goes awry, the entire system is forced to compensate. Even when compensation manages to rescue the most obvious consequences of demyelination, certain processes may not return to a completely normal state, which can lead to problems on longer time scales. The resulting symptoms are a confusing mixture of direct and compensatory changes that continuously evolve. The overall complexity has proven to be intractable to efficient experimental dissection. The application of computational modeling techniques represents an invaluable approach to help break the impasse and engender a new era of understanding and discovery.

## References

[1] Virchow R. (1854). Uber das ausgebreitete Vorkommen einer dem Nervenmark analogen Substanz in den tierischen Geweben. Virchows Arch. Pathol. Anat..

[2] Stiefel K.M., Torben-Nielsen B., Coggan J.S. (2013). Proposed evolutionary changes in the role of myelin. Front. Neurosci..

[3] Bullock T.H., Moore J.K., Fields R.D. (1984). Evolution of myelin sheaths: Both lamprey and hagfish lack myelin. Neurosci. Lett..

[4] Davis A.D., Weatherby T.M., Hartline D.K., Lenz P.H. (1999). Myelin-like sheaths in copepod axons. Nature.

[5] Hartline D.K., Colman D.R. (2007). Rapid conduction and the evolution of giant axons and myelinated fibers. Curr. Biol..

[6] Arancibia-Carcamo I.L., Attwell D. (2014). The node of ranvier in CNS pathology. Acta Neuropathol..

[7] Faisal A.A., White J.A., Laughlin S.B. (2005). Ion-channel noise places limits on the miniaturization of the brain’s wiring. Curr. Biol..

[8] Babbs C.F., Riyi S. (2013). Subtle paranodal injury slows impulse conduction in a mathematical model of myelinated axons. PLoS ONE.

[9] Trapp B.D., Nave K.A. (2008). Multiple sclerosis: An immune or neurodegenerative disorder?. Annu. Rev. Neurosci..

[10] Compston A., Coles A. (2008). Multiple sclerosis. Lancet.

[11] Ostermann P.O., Westerberg C.E. (1975). Paroxysmal attacks in multiple sclerosis. Brain.

[12] Twomey J.A., Espir M.L. (1980). Paroxysmal symptoms as the first manifestations of multiple sclerosis. J. Neurol. Neurosurg. Psychiatry.

[13] Seewann A., Vrenken H., van der Valk P., Blezer E.L., Knol D.L., Castelijns J.A., Polman C.H., Pouwels P.J., Barkhof F., Geurts J.J. (2009). Diffusely abnormal white matter in chronic multiple sclerosis: Imaging and histopathologic analysis. Arch. Neurol..

[14] Ceccarelli A., Bakshi R., Neema M. (2012). MRI in multiple sclerosis: A review of the current literature. Curr. Opin. Neurol..

[15] Moore J.W., Joyner R.W., Brill M.H., Waxman S.D., Najar-Joa M. (1978). Simulations of conduction in uniform myelinated fibers. Relative sensitivity to changes in nodal and internodal parameters. Biophys. J..

[16] Waxman S.G., Kocsis J.D., Stys P.K. (1995). The Axon: Structure, Function and Pathophysiology.

[17] Polman C.H., Reingold S.C., Banwell B., Clanet M., Cohen J.A., Filippi M., Fujihara K., Havrdova E., Hutchinson M., Kappos L. (2011). Diagnostic criteria for multiple sclerosis: 2010 revisions to the McDonald criteria. Ann. Neurol..

[18] Stephanova D.I., Dimitrov B. (2013). Computational Neuroscience: Simulated Demyelinating Neuropathies and Neuronopathies.

[19] Bostock H., Baker M., Reid G. (1991). Changes in excitability of human motor axons underlying post-ischaemic fasciculations: Evidence for two stable states. J. Physiol..

[20] Mogyoros I., Kiernan M.C., Burke D., Bostock H. (1998). Strength-duration properties of sensory and motor axons in amyotrophic lateral sclerosis. Brain.

[21] Kiernan M.C., Burke D., Andersen K.V., Bostock H. (2000). Multiple measures of axonal excitability: A new approach in clinical testing. Muscle Nerve.

[22] Cappelen-Smith C., Kuwabara S., Lin C.S., Mogyoros I., Burke D. (2001). Membrane properties in chronic inflammatory demyelinating polyneuropathy. Brain.

[23] Kuwabara S., Ogawara K., Sung J.Y., Mori M., Kanai K., Hattori T., Yuki N., MLin C.S., Burke D., Bostock H. (2002). Differences in membrane properties of axonal and demyelinating Guillain-Barré syndromes. Ann. Neurol..

[24] Nodera H., Bostock H., Kuwabara S., Sakamoto T., Asanuma K., Jia-Ying S., Ogawara K., Hattori N., Hirayama M., Sobue G. (2004). Nerve excitability properties in Charcot-Marie-Tooth disease type 1A. Brain.

[25] Sung M.H., Simon R. (2004). In silico simulation of inhibitor drug effects on nuclear factor-κB pathway dynamics. Mol. Pharmacol..

[26] Stephanova D.I., Daskalova M. (2005). Differences in potentials and excitability properties in simulated cases of demyelinating neuropathies. Part III. Paranodal internodal demyelination. Clin. Neurophysiol..

[27] Stephanova D.I., Daskalova M.S. (2008). Differences between the channels, currents and mechanisms of conduction slowing/block and accommodative processes in simulated cases of focal demyelinating neuropathies. Eur. Biophys. J..

[28] Stephanova D.I., Alexandrov A.S. (2006). Simulating mild systematic and focal demyelinating neuropathies: Membrane property abnormalities. J. Integr. Neurosci..

[29] Stephanova D.I., Daskalova M., Alexandrov A.S. (2007). Channels, currents and mechanisms of accommodative processes in simulated cases of systematic demyelinating neuropathies. Brain Res..

[30] Stephanova D.I., Krustev S.M., Negrev N., Daskalova M. (2011). The myelin sheath aqueous layers improve the membrane properties of simulated chronic demyelinating neuropathies. J. Integr. Neurosci..

[31] Stephanova D.I., Alexandrov A.S., Kossev A., Christova L. (2007). Simulating focal demyelinating neuropathies: Membrane property abnormalities. Biol. Cybern..

[32] Bø L., Geurts J.J., Mörk S.J., van der Valk P. (2006). Grey matter pathology in multiple sclerosis. Acta Neurol. Scand. Suppl..

[33] Geurts J.J., Barkhof F. (2008). Grey matter pathology in multiple sclerosis. Lancet Neurol..

[34] Zivadinov R., Pirko I. (2012). Advances in understanding gray matter pathology in multiple sclerosis: Are we ready to redefine disease pathogenesis?. BMC Neurol..

[35] Popescu B.F., Lucchinetti C.F. (2012). Pathology of demyelinating diseases. Annu. Rev. Pathol..

[36] Kurtzke J.F., Beebe G.W., Nagler B., Nefzger M.D., Auth T.L., Kurland L.T. (1970). Studies on the natural history of multiple sclerosis: V. Long-term survival in young men. Arch. Neurol..

[37] Rao S.M., Leo G.J., Bernardin L., Unverzagt F. (1991). Cognitive dysfunction in multiple sclerosis. I. Frequency, patterns, and prediction. Neurology.

[38] Meuth S.G., Bittner S., Ulzheimer J.C., Kleinschnitz C., Kieseier B.C., Wiendl H. (2010). Therapeutic approaches to multiple sclerosis: An update on failed, interrupted, or inconclusive trials of neuroprotective and alternative treatment strategies. BioDrugs.

[39] Goldenberg M.M. (2012). Multiple sclerosis review. Pharm. Ther..

[40] Göbel K., Wedell J.H., Herrmann A.M., Wachsmuth L., Pankratz S., Bittner S., Budde T., Kleinschnitz C., Faber C., Wiendl H. (2013). 4-Aminopyridine ameliorates mobility but not disease course in an animal model of multiple sclerosis. Exp. Neurol..

[41] Krishnan A.V., Kiernan M.C. (2013). Sustained-release fampridine and the role of ion channel dysfunction in multiple sclerosis. Mult. Scler..

[42] Bowe C.M., Kocsis J.D., Targ E.F., Waxman S.G. (1987). Physiological effects of 4-aminopyridine on demyelinated mammalian motor and sensory fibers. Ann. Neurol..

[43] Sakurai M., Kanazawa I. (1999). Positive symptoms in multiple sclerosis: Their treatment with sodium channel blockers, lidocaine and mexiletine. J. Neurol. Sci..

[44] Mattson M.P., Guthrie P.B., Kater S.B. (1989). A role for Na^+^-dependent Ca^2+^ extrusion in protection against neuronal excitotoxicity. FASEB J..

[45] Moll N.M., Rietsch A.M., Thomas S., Ransohoff A.J., Lee J.C., Fox R., Chang A., Ransohoff R.M., Fisher E. (2011). Multiple sclerosis normal-appearing white matter: Pathology-imagig correlations. Ann. Neurol..

[46] Lucchinetti C., Brück W., Parisi J., Scheithauer B., Rodriguez M., Lassmann H. (2000). Heterogeneity of multiple sclerosis lesions: Implications for the pathogenesis of demyelination. Ann. Neurol..

[47] Kassmann C.M., Nave K.A. (2008). Oligodendroglial impact on axonal function and survival— A hypothesis. Curr. Opin. Neurol..

[48] Scolding N., Franklin R. (1998). Axon loss in multiple sclerosis. Lancet.

[49] Mi S., Miller R.H., Lee X., Scott M.L., Shulag-Morskaya S., Shao Z., Chang J., Thill G., Levesque M., Zhang M. (2005). LINGO-1 negatively regulates myelination by oligodendrocytes. Nat. Neurosci..

[50] Bittner S., Ruck T., Schuhmann M.K., Herrmann A.M., Maati H.M., Bobak N., Göbel K., Langhauser F., Stegner D., Ehling P. (2013). 2013 Endothelial TWIK-related potassium channel-1 (TREK1) regulates immune-cell trafficking into the CNS. Nat. Med..

[51] Viglietta V., Baecher-Allan C., Weiner H.L., Hafler D.A. (2004). Loss of functional suppression by CD4^+^CD25^+^ regulatory T cells in patients with multiple sclerosis. J. Exp. Med..

[52] Zajicek J.P., Wing M., Scolding N.J., Compston D.A. (1992). Interactions between oligodendrocytes and microglia. A major role for complement and tumour necrosis factor in oligodendrocyte adherence and killing. Brain.

[53] Nikić I., Merkler D., Sorbara C., Brinkoetter M., Kreutzfeldt M., Bareyre F.M., Brück W., Bishop D., Misgeld T., Kerschensteiner M. (2011). A reversible form of axon damage in experimental autoimmune encephalomyelitis and multiple sclerosis. Nat. Med..

[54] Barnett M.H., Prineas J.W. (2004). Relapsing and remitting multiple sclerosis: Pathology of the newly forming lesion. Ann. Neurol..

[55] Van der Laan L.J., Ruuls S.R., Weber K.S., Lodder I.J., Döpp E.A., Dijkstra C.D. (1996). Macrophage phagocytosis of myelin *in vitro* determined by flow cytometry: Phagocytosis is mediated by CR3 and induces production of tumor necrosis factor-α and nitric oxide. J. Neuroimmunol..

[56] Wang G., Thompson S.M. (2008). Maladaptive homeostatic plasticity in a rodent model of central pain syndrome: Thalamic hyperexcitability after spinothalamic tract lesions. J. Neurosci..

[57] Coggan J.S., Prescott S.A., Bartol T.M., Sejnowski T.J. (2010). Imbalance of ionic conductances contributes to diverse symptoms of demyelination. Proc. Natl. Acad. Sci. USA.

[58] Coggan J.S., Ocker G.K., Sejnowski T.J., Prescott S.A. (2011). Explaining pathological changes in axonal excitability through dynamical analysis of conductance-based models. J. Neural Eng..

[59] Coggan J.S., Prescott S.A., Sejnowski T.J. (2015). Cooperativity between remote sites of ectopic spiking allows afterdischarge to be initiated and maintained at different locations. J. Comput. Neurosci..

[60] Aon M.A., Cortassa S., Akar F.G., Brown D.A., Zhou L., O’Rourke B. (2009). From mitochondrial dynamics to arrhythmias. Int. J. Biochem. Cell Biol..

[61] Su K., Bourdette D., Forte M. (2013). Mitochondrial dysfunction and neurodegeneration in multiple sclerosis. Front. Physiol..

[62] Soltesz I., Staley K. (2008). Computational Neuroscience in Epilepsy.

[63] Hodgkin A.L., Huxley A.F. (1952). The components of membrane conductance in the giant axon of *Loligo*. J. Physiol..

[64] Hodgkin A.L., Huxley A.F. (1952). Currents carried by sodium and potassium ions through the membrane of the giant axon of *Loligo*. J. Physiol..

[65] Fitzhugh R. (1962). Computation of impulse initiation and saltatory conduction in a myelinated nerve fiber. Biophys. J..

[66] Goldman L., Albus J.S. (1968). Computation of impulse conduction in myelinated fibers; theoretical basis of the velocity-diameter relation. Biophys. J..

[67] Frankenhaeuser B., Huxley A.F. (1964). The action potential in the myelinated nerve fiber of *Xenopus*
*laevis* as computed on the basis of voltage clamp data. J. Physiol..

[68] Smith R.S., Koles Z.J. (1970). Myelinated nerve fibers: Computed effect of myelin thickness on conduction velocity. Am. J. Physiol..

[69] Hutchinson N.A., Koles Z.J., Smith R.S. (1970). Conduction velocity in myelinated nerve fibres of *Xenopus*
*laevis*. J. Physiol..

[70] Koles Z.J., Rasminsky M. (1972). A computer simulation of conduction in demyelinated nerve fibres. J. Physiol..

[71] Hardy W.L. (1973). Propagation speed in myelinated nerve. II. Theoretical dependence on external Na and on temperature. Biophys. J..

[72] Schauf C.L., Davis F.A. (1974). Impulse conduction in multiple sclerosis: A theoretical basis for modification by temperature and pharmacological agents. J. Neurol. Neurosurg. Psychiatry.

[73] Brill M.H., Waxman S.G., Moore J.W., Joyner R.W. (1977). Conduction velocity and spike configuration in myelinated fibres: Computed dependence on internode distance. J. Neurol. Neurosurg. Psychiatry.

[74] Waxman S.G., Brill M.H. (1978). Conduction through demyelinated plaques in multiple sclerosis: Computer simulations of facilitation by short internodes. J. Neurol. Neurosurg. Psychiatry.

[75] Wood S.L., Waxman S.G., Kocsis J.D. (1982). Conduction of trans of impulses in uniform myelinated fibers: Computed dependence on stimulus frequency. Neuroscience.

[76] Goldfinger M.D. (2000). Computation of high safety factor impulse propagation at axonal branch points. Neuroreport.

[77] Blight A.R. (1985). Computer simulation of action potentials and afterpotentials in mammalian myelinated axons: The case for a lower resistance myelin sheath. Neuroscience.

[78] Halter J.A., Clark J.W. (1991). A distributed-parameter model of the myelinated nerve fiber. J. Theor. Biol..

[79] Schwarz J.R., Eikhof G. (1987). Na currents and action potentials in rat myelinated nerve fibres at 20 and 37 °C. Pflugers Arch..

[80] Stephanova D.I. (2001). Myelin as longitudinal conductor: A multi-layered model of the myelinated human motor nerve fibre. Biol. Cybern..

[81] McIntyre C.C., Richardson A.G., Grill W.M. (2002). Modeling the excitability of mammalian nerve fibers: Influence of afterpotentials on the recovery cycle. J. Neurophysiol..

[82] Einziger P.D., Livshitz L.M., Mizrahi J. (2005). Generalized cable equation model for myelinated nerve fiber. IEEE Trans. Biomed. Eng..

[83] Volman V., Ng L. (2014). Primary paranode demyelination modulates slowly developing axonal depolarization in a model of axonal injury. J. Neural Comput..

[84] Stephanova D.I., Bostock H. (1995). A Distributed-parameter model of the myelinated human motor nerve fibre: Temporal and spatial distributions of action potentials and ionic currents. Biol. Cybern..

[85] Chiu S.Y., Ritchie J.M. (1984). On the physiological role of internodal potassium channels and the security of conduction in myelinated nerve fibres. Proc. R. Soc. Lond. B Biol. Sci..

[86] Brismar T., Schwarz J.R. (1985). Potassium permeability in rat myelinated nerve fibres. Acta Physiol. Scand..

[87] Chiu S.Y., Schwarz W. (1987). Sodium and potassium currents in acutely demyelinated internodes of rabbit sciatic nerves. J. Physiol..

[88] Baker M., Bostock H., Grafe P., Martius P. (1987). Function and distribution of three types of rectifying channel in rat spinal root myelinated axons. J. Physiol..

[89] Röper J., Schwarz J.R. (1989). Heterogeneous distribution of fast and slow potassium channels in myelinated rat nerve fibres. J. Physiol..

[90] Bittner S., Meuth S.G. (2013). Targeting ion channels for the treatment of autoimmune neuroinflammation. Ther. Adv. Neurol. Disord..

[91] Waxman S.G., Ritchie J.M. (1993). Molecular dissection of the myelinated axon. Ann. Neurol..

[92] Bittner S., Budde T., Wiendl H., Meuth S.G. (2010). From the background to the spotlight: TASK channels in pathological conditions. Brain Pathol..

[93] Ehling P., Bittner S., Budde T., Wiendl H., Meuth S.G. (2011). Ion channels in autoimmune neurodegeneration. FEBS Lett..

[94] Hübel N., Dahlem M.A. (2014). Dynamics from seconds to hours in Hodgkin-Huxley model with time-dependent ion concentrations and buffer reservoirs. PLoS Comput. Biol..

[95] Ganapathy L., Clark J.W. (1987). Extracellular currents and potentials of the active myelinated nerve fibre. Biophys. J..

[96] Prescott S.A., Jaeger D., Jung R. (2015). Pathological changes in peripheral nerve excitability. Encyclopedia of Computational Neurosci.

[97] Barrett E.F., Barrett J.N. (1982). Intracellular recording from vertebrate myelinated axons: Mechanism of the depolarizing afterpotential. J. Physiol..

[98] Blight A.R., Someya S. (1985). Depolarizing afterpotentials in myelinated axons of mammalian spinal cord. Neuroscience.

[99] Richardson A.G., McIntyre C.C., Grill W.M. (2000). Modelling the effects of electric fields on nerve fibres: Influence of the myelin sheath. Med. Biol. Eng. Comput..

[100] Zlochiver S. (2010). Persistent reflection underlies ectopic activity in multiple sclerosis: A numerical study. Biol. Cybern..

[101] Ratté S., Zhu Y., Lee K.Y., Prescott S.A. (2014). Criticality and degeneracy in injury-induced changes in primary afferent excitability and the implications for neuropathic pain. Elife.

[102] Zhu Y., Feng B., Schwartz E.S., Gebhart G.F., Prescott S.A. (2015). Novel method to assess axonal excitability using channelrhodopsin-based photoactivation. J. Neurophysiol..

[103] Tagoe T., Barker M., Jones A., Allcock N., Hamann M. (2014). Auditory nerve perinodal dysmyelination in noise-induced hearing loss. J. Neurosci..

[104] Brown A.M., Hamann M. (2014). Computational modeling of the effects of auditory nerve dysmyelination. Front. Neuroanat..

[105] Stephanova D.I., Daskalova M. (2015). Electrotonic potentials in simulated chronic inflammatory demyelinating polyneuropathy at 20 °C–42 °C. J. Integr. Neurosci..

[106] Rasminsky M. (1981). Hyperexcitability of pathologically myelinated axons and positive symptoms in multiple sclerosis. Adv. Neurol..

[107] Ulrich J., Groebke-Lorenz W. (1983). The optic nerve in multiple sclerosis: A morphological study with retrospective clinicopathological correlation. Neuro-Ophthalmology.

[108] Hamada M.S., Kole M.H. (2015). Myelin loss and axonal ion channel adaptations associated with gray matter neuronal hyperexcitability. J. Neurosci..

[109] Yu N., Morris C.E., Joós B., Longtin A. (2012). Spontaneous excitation patterns computed for axons with injury-like impairments of sodium channels and Na/K pumps. PLoS Comput. Biol..

[110] Lopreore C.L., Bartol T.M., Coggan J.S., Keller D.X., Sosinsky G.E., Ellisman M.H., Sejnowski T.J. (2008). Computational modeling of three-dimensional electrodiffusion in biological systems: Application to the node of Ranvier. Biophys. J..

[111] Broome T.M., Cole.man R.A. (2011). A mathematical model of cell death in multiple sclerosis. J. Neurosci. Methods.

[112] Ridsdale R.A., Beniac D.R., Tompkins T.A., Moscarello M.A., Harauz G. (1997). Three-dimensional structure of myelin basic protein. II. Molecular modeling and considerations of predicted structures in multiple sclerosis. J. Biol. Chem..

[113] Pigozzo A.B., Macedo G.C., Santos R.W., Lobosco M. (2013). On the computational modeling of the innate immune system. BMC Bioinform..

[114] Doerck S., Göbel K., Weise G., Schneider-Hohendorf T., Reinhardt M., Hauff P., Schwab N., Linker R., Mäurer M., Meuth S.G. (2010). Temporal pattern of ICAM-I mediated regulatory T cell recruitment to sites of inflammation in adoptive transfer model of multiple sclerosis. PLoS ONE.

[115] De Mendizábal N.V., Carneiro J., Solé R.V., Goñi J., Bragard J., Martinez-Forero I., Martinez-Pasamar S., Sepulcre J., Torrealdea J., Bagnato F. (2011). Modeling the effector-regulatory T cell cross-regulation reveals the intrinsic character of relapses in Multiple Sclerosis. BMC Syst. Biol..

[116] Jolivet R., Coggan J.S., Allaman I., Magistretti P.J. (2015). Multi-timescale modeling of activity-dependent metabolic coupling in the neuron-glia-vasculature ensemble. PLoS Comput. Biol..

[117] Zeis T., Allaman I., Gentner M., Schroder K., Tschopp J., Magistretti P.J., Schaeren-Wiemers N. (2015). Metabolic gene expression changes in astrocytes in Multiple Sclerosis cerebral cortex are indicative of immune-mediated signaling. Brain Behav. Immun..

